# Supplementary material for: Mitochondria-Targeted Antioxidant SkQ1 Prevents the Development of Experimental Colitis in Mice and Impairment of the Barrier Function of the Intestinal Epithelium
Source: Cells. 2022 Oct 31;11(21):3441. doi: 10.3390/cells11213441 (PMC9659222; doi:10.3390/cells11213441)
Supplement: Supplementary file 1 [file cells-11-03441-s001.zip › cells-1960994-supplementary.pdf]

Supplementary Table S1. Histological grading of colitis

Feature	Score	Description
Damage	0	**None**
	1	**Loss of the basal ⅓ of the crypt**
	2	**Loss of the basal ⅔ of the crypt**
	3	**Loss of entire crypt but intact surface epithelial cells**
	4	**Loss of both the entire crypt and the surface epithelial cells**
Extension	0	**None**
	1	**Focal**
	2	**Lesions involving ⅓ of the intestine**
	3	**Lesions involving ⅔ of the intestine**
	4	**Lesions involving the entire intestine**

Supplementary Table S2. Primer sequences used in this study.

Gene	Forward primer (5'-3')	Reverse primer (5'-3')
EEF2	TGTCAGTCATCGCCCATGTG	CATCCTTGCGAGTGTCAGTGA
HO1	CACGCATATACCCGCTACC	TCATCTCCAGAGTGTTCATTCG
ICAM1	CAGGTATACAAGTTACAGAAGG	TGACAGCCAGAGGAAGTG
IL-18	GTTCACTCTCATAACTTACATCAA	ATACAGGCGAGGTCATCAC
IL-1α	AGACTCTACACATATTACAG	CAGCATTAAGTTCTTATACC
IL-1β	TCAATGGACAGAATATCAAC	CACAGGACAGGTATAGATT
IL-6	ACCGCTATGAAGTTCCTCTC	CTCTGTGAAGTCTCCTCTCC
IL-8	ACTTCAAGAACATCCAGAGC	CTTTCCAGGTCAGTTAGCC
TBP	ACCGTGAATCTTGGCTGTAAAC	GCAGCAAATCGCTTGGGATTA
TNFα	CGTGGAACTGGCAGAAGAG	ACAAGCAGGAATGAGAAGAGG

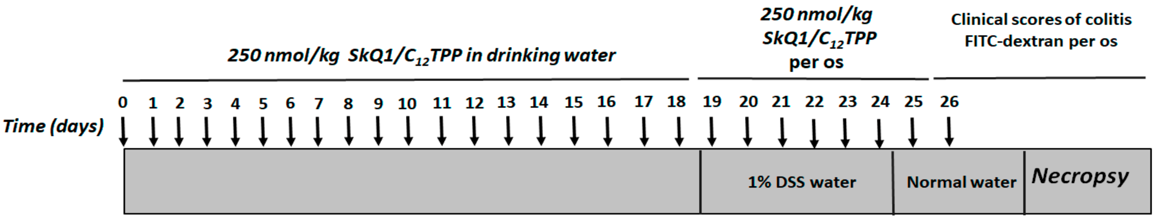

Supplementary Figure S1. Experimental design for DSS-induced colitis in mice. Two groups of mice received SkQ1 and C_{12}TPP for 18 days with drinking water and per os from day 19 to 26. To induce colitis, mice were given 1% DSS in drinking water from day 19 to 24, then DSS was changed to tap water for 1 day. On day 8, the clinical assessment of colitis was examined. Mice received 4 kDa FITC-dextran per os. After 4 hours, mice were anesthetized and blood was taken from the jugular veins for further measurement of fluorescence in plasma. The colons were removed, measured, and used for further histological and gene expression analysis.

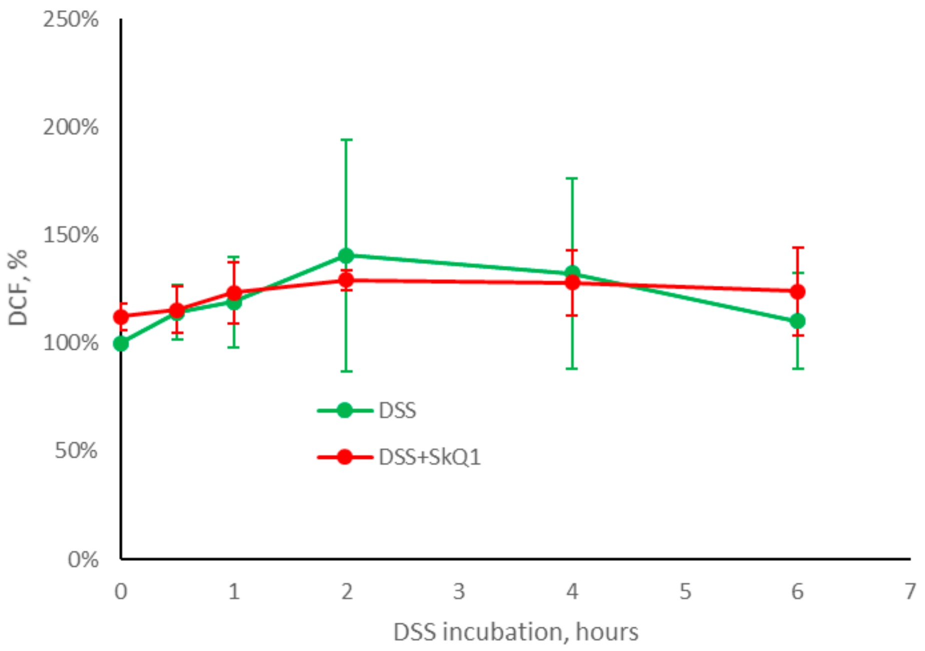

Supplementary Figure S2. Effect of SkQ1 on intracellular ROS level in Caco-2 intestinal cells after DSS treatment. Intracellular ROS were measured as DCFH2-DA oxidation with flow cytometry. After treatment with 2 nM of SkQ1 for 48 h cells were treated with 2% DSS for indicated time. Thereafter they were loaded with 5 μM DCFH2-DA for 20 min at 37C. Flow cytometric analysis was performed using flow cytometer Beckman Coulter FC500. Results are expressed as mean ± SD (n=3).
